# Sensory Substitution: The Spatial Updating of Auditory Scenes “Mimics” the Spatial Updating of Visual Scenes

**DOI:** 10.3389/fnbeh.2016.00079

**Published:** 2016-04-21

**Authors:** Achille Pasqualotto, Tayfun Esenkaya

**Affiliations:** ^1^Faculty of Arts and Social Sciences, Sabanci UniversityIstanbul, Turkey; ^2^Department of Psychology, University of BathBath, UK

**Keywords:** sensory substitution, vOICe, allocentric, egocentric, perspective-taking

## Abstract

Visual-to-auditory sensory substitution is used to convey visual information through audition, and it was initially created to compensate for blindness; it consists of software converting the *visual* images captured by a video-camera into the equivalent *auditory* images, or “soundscapes”. Here, it was used by blindfolded sighted participants to learn the spatial position of simple shapes depicted in images arranged on the floor. Very few studies have used sensory substitution to investigate *spatial* representation, while it has been widely used to investigate object *recognition*. Additionally, with sensory substitution we could study the performance of participants *actively* exploring the environment through audition, rather than *passively* localizing sound sources. Blindfolded participants *egocentrically* learnt the position of six images by using sensory substitution and then a judgment of relative direction task (JRD) was used to determine how this scene was represented. This task consists of *imagining* being in a given location, oriented in a given direction, and pointing towards the required image. Before performing the JRD task, participants explored a map that provided *allocentric* information about the scene. Although spatial exploration was egocentric, surprisingly we found that performance in the JRD task was better for allocentric perspectives. This suggests that the egocentric representation of the scene was *updated*. This result is in line with previous studies using visual and somatosensory scenes, thus supporting the notion that different sensory modalities produce equivalent spatial representation(s). Moreover, our results have practical implications to improve training methods with sensory substitution devices (SSD).

## Introduction

Knowing the location of external objects is critical for survival, and in many species this function depends on vision. In case of visual loss, participants involved in spatial tasks showed that the non-visual modalities could partially (but not fully) compensate for the lack of visual input (Putzar et al., 2007; King, 2009; Gori et al., 2010, 2014; Papadopoulos et al., 2012; Pasqualotto and Proulx, 2012). Among the tools created to compensate for visual loss, sensory substitution has provided excellent practical and theoretical results (Bach-y-Rita et al., 1969; Bach-y-Rita, 1972; Proulx et al., 2014b). For example, it shed light on the mechanisms of neural plasticity in both visually impaired *and* sighted individuals (Rauschecker, 1995; Sampaio et al., 2001; Bach-y-Rita and Kercel, 2003; Tyler et al., 2003; Amedi et al., 2007; Proulx et al., 2014a; Brown et al., 2015). Sensory substitution devices (SSD) convey visual information through other modalities, thus allowing for recognition of distant and silent objects. In visual-to-auditory sensory substitution, software called “The vOICe” converts the images^1^ captured by a video-camera (controlled by the user) into equivalent “soundscapes” (or auditory images), which is listened through headphones. To transform visual images into auditory images, the software scans visual images from left-to-right, converts them into grayscale images, and subdivides them into pixels; each pixel is then converted into sound (or “sonified”) based on its luminance, horizontal position, and vertical position. High luminance pixels will sound *louder* than low luminance pixels, pixels on the left will be played *before* than those on the right, and pixels at the top will have a higher *pitch* than those at the bottom. Thus, visual images are sonified by using three parameters: loudness, time, and pitch (Meijer, 1992). Another feature of sensory substitution is that the active movement of the camera performed by the users of a SSD is crucial for the successful recognition of the objects (White et al., 1970; Bach-y-Rita, 1972; Lenay et al., 2003). Thus, in our study participants explored the environment by using actively moving the video-camera.

There are many studies using sensory substitution that are concerned with *recognition* tasks (i.e., “what” tasks; Ptito et al., 2005; Kim and Zatorre, 2008; Brown et al., 2011; Striem-Amit et al., 2012; Haigh et al., 2013). However, studies using The vOICe in *spatial* tasks (i.e., “where” tasks) remain sparse, and more research is needed in order to improve training regimens with SSD (Auvray et al., 2007; Proulx et al., 2008; Chebat et al., 2011). As a matter of fact, lengthy and frustrating training with SSD has been blamed for the scarce utilization of these tools (included The vOICe) in everyday life (Loomis, 2010; Maidenbaum et al., 2014). Although in some cases the input from SSD can be successfully interpreted by naïve users (Auvray et al., 2005; Stiles et al., 2015), usually training is required to perform most tasks employing SSD (Bach-y-Rita, 1972; Meijer, 1992). In the present study, before the main experiment, participants were trained to recognize simple images by using The vOICe.

Unlike the studies investigating auditory spatial representation where participants *passively* listened to sounds delivered by different sources (e.g., Klatzky et al., 2003), using The vOICe allowed for studying *active* spatial exploration (Auvray et al., 2005; Stiles et al., 2015). Therefore, participants actively explored the environment, which included the target images and environmental features such as the floor, thus providing novel insights on active spatial exploration performed through audition (Klatzky et al., 1998; Gaunet et al., 2001). Finally, the use of a visual-to-auditory sensory substitution device was necessary because audition is ill-suited for exploring silent objects (Yamamoto and Shelton, 2009; Avraamides and Kelly, 2010).

In this study we investigated how an auditory-learnt scene (multiple objects) was represented. There are two major manners to represent spatial information; egocentrically, where the spatial relations between the observer’s position and the position of each object are stored in spatial memory; or allocentrically, where the spatial relations among the observed objects are stored in spatial memory (McNamara, 2003). Our purpose is to use The vOICe (i.e., auditory input) to investigate how a regularly arranged scene (“chessboard-like” arrangement) is stored in spatial memory. Previous studies using vision demonstrated that, rather than being represented according to the egocentric “viewpoint”^2^ (Mou and McNamara, 2002), regularly arranged scenes were represented according to the reference frame used during scene learning. In fact, when observers learnt the scene egocentrically, the resulting spatial representation was egocentric; contrarily, when observers learnt the scene allocentrically, the resulting spatial representation was allocentric (see also Wolbers and Büchel, 2005; Pasqualotto and Proulx, 2013; Pasqualotto et al., 2013a; Thibault et al., 2013). Additionally, once spatial representation is formed, it can be *updated* by subsequent input (Simons and Wang, 1998; Mou et al., 2004). Spatial updating has been extensively studied for visually-learnt (Diwadkar and McNamara, 1997; Simons and Wang, 1998; Zhao et al., 2007) and haptically-learnt scenes (Newell et al., 2005; Pasqualotto et al., 2005). However, there is little research on auditory-learnt spatial updating (Loomis et al., 2002; Klatzky et al., 2003).

The method used in this article will be based on the study by Pasqualotto et al. (2013b), where groups of participants with different levels of visual experience (but here we will focus on blindfolded sighted) egocentrically learnt a regularly arranged scene through somatosensation; they repeatedly walked from the “viewpoint” to each object composing the scene. After the egocentric learning, participants received allocentric information about the scene (a map disclosing the regular structure of the scene, but *not* the actual objects) to investigate whether spatial updating could take place. The resulting spatial representation was investigated by a perspective-taking task (or JRD). For those unfamiliar with this task, it involves aligning themselves with an imagery perspective and pointing to the required object/landmark (Mou and McNamara, 2002; McNamara, 2003). For example, imagine that you are in your kitchen near the fridge, that you are looking towards the sink (imaginary perspective), and that you have to point towards the kitchen door (required object). Pasqualotto et al. (2013b) found that the pointing performance of blindfolded sighted participants showed the characteristic saw-tooth profile (e.g., Diwadkar and McNamara, 1997; Shelton and McNamara, 2001; Mou and McNamara, 2002), where allocentric perspectives were better performed than egocentric ones, thus suggesting that spatial updating occurred (i.e., the egocentric representation of the scene was updated into an allocentric representation). In particular, Mou and McNamara (2002) found that the representation of a regularly arranged scene was based on the allocentric structure of the scene (i.e., spatial relations among objects) rather than on the experienced view (egocentric). In place of somatosensation, here blindfolded sighted participants used audition (i.e., the SSD) to learn the spatial location of six images.

In case the present study will replicate the results by Pasqualotto et al. (2013b), this would suggest that auditory-learnt scenes are represented in an equivalent manner to those learnt by vision and somatosensation (Giudice et al., 2011; Loomis et al., 2013; Intraub et al., 2015). Taking into consideration that, independently from the sensory modality, representing the space is subserved by the same brain areas (Kandel et al., 2012), we expect to replicate the findings by Pasqualotto et al. (2013b).

## Materials and Methods

### Participants

Eighteen sighted participants (nine male) recruited among the students of the Sabanci University participated in the experiment. Their average age was 22.4 years. No participant suffered from hearing/motor impairments and all signed the informed consent form. This study was carried out in accordance with the recommendations of Declaration of Helsinki and the protocol was approved by the Sabanci University Research Ethics Committee. Participants received meal-vouchers for their participation. Each participant went to the lab three times across three consecutive days (once per day).

### Apparatus

#### Apparatus Training Sessions

**Figure 1:**
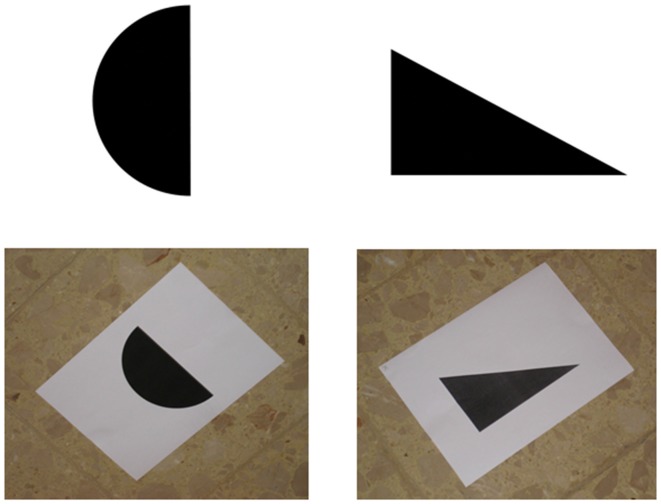
**Examples of the images used in the first (the two at the top) and the second training day (the two at the bottom)**.

**Figure 2:**
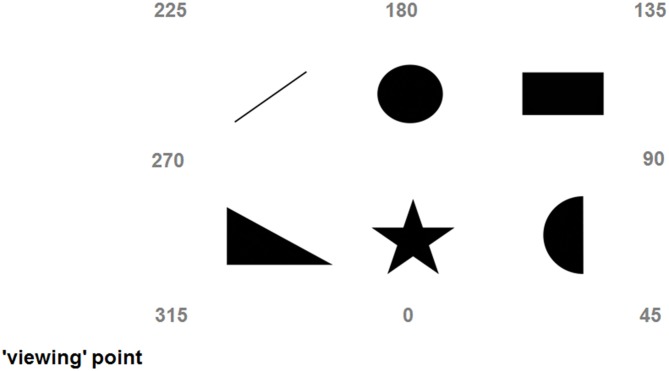
**Depiction of the scene explored during the third day (main experiment); numbers indicate the imaginary headings/perspectives (315, 45, 135, and 225 are egocentric, while 0, 90, 180, and 270 are allocentric)**.

During the 2 days preceding the main experiment, participants familiarized with the six images that were going to be used during the main experiment (a triangle, a star, a moon crescent, a rectangle, a circle, and an upward bar). Although there is evidence that untrained participants can recognize sonified images (Auvray et al., 2005; Stiles et al., 2015), the use of SSD usually requires some amount of training (Bach-y-Rita, 1972; Meijer, 1992). In fact, without training our participants would have been completely confused and helpless. Microsoft^™^ PowerPoint presentations were used to train participants. For sake of clarity and simplicity, during the first training day the six images were presented upright and on a white background (see the top part of Figure 1). During the second training day the same six images were presented as they were going to be “seen” during the main experiment, that is, printed on a white sheet laying on a “patterned floor” (i.e., the floor of the room where the main experiment took place). In fact, the soundscape generated by The vOICe includes all the items that in a given moment are captured by the video-camera, including the target image, the sheet, and the surrounding floor. Hence, we trained participants to recognize sonified images embedded in the “noise” produced by the sonified sheet and floor (see the bottom part of Figure 1). Additionally, in the second training day images were presented as participants were going to “see” them from the “viewing point” during the main experiment (i.e., tilted, see the bottom part of Figure 1). To better understand this point, you can look at Figure 2, imagine standing at the viewing point, and realize that from there images are seen as in the bottom part of Figure 1.

#### Apparatus Main Experiment

During the main experiment the six familiar images were arranged on the floor of a large room (about 5 m by 4 m), with 60 cm distance between any two of them (see Figure 2). Each image was printed in black-and-white on A4 sheets. The settings of The vOICe (freely available at: www.seeingwithsound.com) were: standard view, ×2 zoom, and medium loudness. The perspective-taking task was conducted in a different room (walking there took about 2 min) using a LogiTech^™^ 3DPro Joystick connected to a HP^™^ desktop running a MatLab^™^ program. There were 40 trials where participants *imagined* aligning themselves to eight perspectives. Four of these imaginary perspectives (20 trials in total) were called “egocentric” and consisted of the subjective perspective of the scene (i.e., the “view” from the “viewing” point, 315°), its mirror perspective (i.e., the “view” from the opposite side of the scene, 135°) and the two intermediate perspectives (45° and 225°). For example, imagining being near the triangle and facing the circle would be a 315° perspective, while imagining being near the circle and facing the triangle would be a 135° perspective (see Figure 2). The remaining four imaginary perspectives (20 trials in total) were called “allocentric” and were the perspectives aligned with the intrinsic axes of the scene (0°, 90°, 180°, and 270°). For example, imagining being near the triangle and facing the upward bar would be a 0° perspective, while imagining being near the upward bar and facing the triangle would be a 180° perspective (see Figure 2; Pasqualotto et al., 2013b). In sum, egocentric perspectives were those spatially related with the subjective view of the scene, while allocentric perspectives were those spatially related with the intrinsic axes of the scene. For sake of consistency and clarity, the labels associated to the perspectives were the same as Mou and McNamara (2002) and Pasqualotto et al. (2013b); that is, we could have labeled the subjective perspective experienced by the participants “0°” rather than “315°” and have all the other perspectives renamed (or even Ego1, Allo1, Ego2, Allo2, etc.), but we preferred to use the same names for easier comparison. During the perspective-taking task participants imagined being aligned to one of the eight perspectives and had to point to the required object (e.g., “Imagine that you are near the triangle and that you are facing the upward bar, point to the star”; pointing the joystick to create a 90° angle would be the best answer). Therefore, the way participants performed this perspective-taking task would inform us about which spatial representation of the scene (egocentric/allocentric) they possessed.

### Procedure

#### Procedure Training Sessions

Training was necessary so that, during the main experiment, participants would have been able to actively explore a meaningful scene. In other words, participants would have been able to recognize the images and form a spatial representation of the scene. On the first training day participants signed the consent form and familiarized themselves with the six images that were used in the main experiment. Participants were initially presented with each visual image coupled with its soundscapes (e.g., a triangle coupled with the soundscape of that triangle). Once participants were sufficiently confident (i.e., after about 3–4 repetitions), they were presented with the soundscapes alone, and were asked to declare to which visual image they corresponded (e.g., when the soundscape of a triangle was played then participants were expected to answer: “Triangle!”). The training terminated after two consecutive error-free runs (on average after 5–6 runs). The first training session lasted for about 20 min.

The day after, participants underwent the second training session. Here each image was presented (both visually and auditorily) printed on a white sheet and with the floor of the room where the main experiment was going to take place. Additionally, images were presented tilted because this is how they were going to be seen from the “viewing point” (as abovementioned, see bottom part of Figures 1, 2 to understand this point). This training was necessary to ensure that, during the main experiment, participants would have been able to recognize the images by audition alone. The second training session proceeded as the first one; presentation and testing that ended after two consecutive runs without errors (on average after 4–5 runs). Finally, in this session participants familiarized themselves with the use of the joystick for the JRD task. Here, they were asked to use the joystick to point towards well-known locations inside the campus (e.g., “Imagine that you are at the main gate, that you are facing the library, point to the bus station”). During the main experiment, the JRD was performed by using the six objects. The second training session lasted about 25 min.

#### Procedure Main Experiment

On the third consecutive day, participants run the main experiment; they were blindfolded and guided to the room where the six printed images were set on the floor (see Figure 2). Blindfolded participants were asked to wear headphones to listen to the soundscapes generated by The vOICe. They were instructed to stand still and were oriented along the 315° viewpoint (see Figure 2). To familiarize blindfolded participants with the place where they were, initially they were instructed to use the video-camera to explore the environment itself (walls, floor^3^, ceiling, doors, etc.), and only then the six images arranged on the floor. Blindfolded participants were guided twice by the experimenter to each image by following the serial learning sequence: triangle, star, moon, rectangle, circle, and upward bar. This procedure was aimed to trigger the use of an egocentric reference frame (Pasqualotto et al., 2013b) and, by using a ×2 zoom, we ensured that images were “seen” one-by-one only. Thus, information on the spatial relations *among* the images was not accessible. In fact, this procedure emphasized the spatial relationships among each image and the “observer” (i.e., egocentric scene representation). During this guided learning phase, blindfolded participants had to correctly name each image. Then for about 2 min participants explored the images without guidance by following the same sequence and by naming each object.

**Figure 3:**
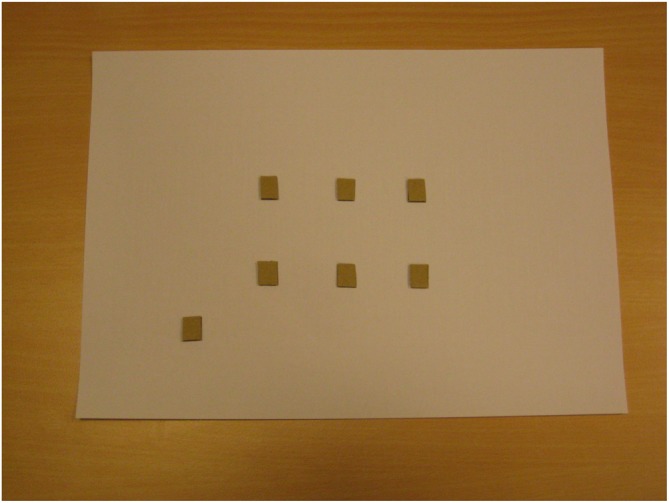
**The “map” explored by blindfolded participants; it “shows” (no vision involved) the regular structure of the scene without disclosing the identity of the images (triangle, circle, etc.).** The lower-left dot represents the point where participants stood.

Subsequently, blindfolded participants were brought to the room where the perspective-taking task was performed. Before starting the task, blindfolded participants explored a map showing a bird-eye view of the regularly arranged scene (six dots), in addition to a seventh dot representing the point where blindfolded participants stood during scene exploration (“viewing” point). Blindfolded participants were told that the map represented the scene and that each dot represented one image (the identity of the images was not disclosed); in fact, the map reported seven identical raised dots (see Figure 3). By providing allocentric (or configurational) information, this procedure was aimed to trigger the spatial updating of the scene (see Pasqualotto et al., 2013b). Blindfolded participants used both hands to explore the map; initially they were assisted by the experimenter and then they explored the map on their own. Map exploration with the experimenter (1 min) proceeded along the same serial learning sequence followed during the exploration of the *real* scene, while unassisted exploration (1 min) was unconstrained.

The perspective-taking task consisted of 40 randomized trials, 20 requiring blindfolded participants to imagine perspectives related with the subjective “view” of the scene (egocentric) and 20 related with the intrinsic axes of the scene (allocentric). The experimenter read out each trial for the blindfolded participants, for example: “Imagine that you are near the star (3-s pause), facing the rectangle (3-s pause), point to the circle!” or “Imagine that you are near the rectangle (3-s pause), facing the circle (3-s pause), point to the moon!” (see Pasqualotto et al., 2013b). An auditory cue (a “bling” sound) was played to prompt blindfolded participants’ responses (i.e., joystick aiming). In the end, participants took off the blindfold and were debriefed. Pointing errors in degrees (°) and reaction times (ms) were recorded. The main experiment took about 40 min.

## Results

**Figure 4:**
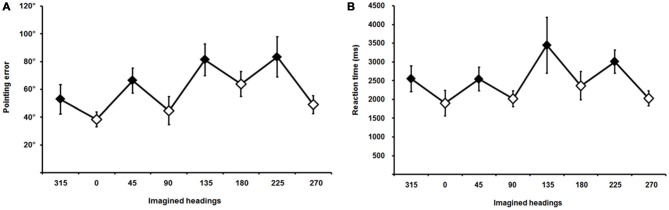
**Pointing errors (A) and reaction times (B) across different imaginary headings or perspectives; 315, 45, 135, and 225 (filled diamonds) are the egocentric and 0, 90, 180, and 270 (empty diamonds) are the allocentric perspectives.** Error bars represent the ±SE.

Average pointing errors and reaction times are plotted in Figure 4; they showed the classic saw-tooth pattern (Mou and McNamara, 2002; Pasqualotto et al., 2013b), indicating that trials involving allocentric perspectives were performed more accurately (smaller pointing errors) and more rapidly (shorter reaction times) than trials involving egocentric perspectives. Pointing errors were normally distributed^4^, thus we started by analyzing their main effect across the eight imaginary perspectives (average pointing errors for each perspective and for each participant) by using one-way ANOVA; results showed that existed significant differences across the perspectives [*F*_(1,17)_ = 5.41, *p* = 0.001].

Thus, we continued the analysis with a paired-samples *t*-test comparing average pointing errors for egocentric vs. allocentric perspectives for each participant, which showed a significant effect [*t*_(17)_ = −4.04, *p* = 0.001] indicating that participants’ performance in the JRD task was more accurate when the imaginary perspectives were aligned with the allocentric axes of the scene (0°, 90°, 180°, and 270°) than with the egocentric perspectives of the scene (45°, 135°, 225°, and 315°). Respectively, average pointing errors were 49.04° (standard deviation 10.82) and 71.03° (SD 14.26; see Figure 4A).

Reaction times were normally distributed^5^ and, as we did for pointing errors, average reaction times across the eight imaginary perspectives were initially analyzed by using one-way ANOVA; results supported the existence of a significant difference across perspectives [*F*_(1,17)_ = 2.93, *p* = 0.01].

The paired-samples *t*-test performed on the average reaction times showed a significant effect of the imaginary perspective [*t*_(17)_ = 2.84, *p* = 0.01], indicating that participants pointed to the targets more rapidly when imaginary perspectives were aligned with the allocentric axes of the scene (average reaction time 2078 ms, SD 1235) than when aligned with the egocentric views (average reaction time 2886 ms, SD 1980; see Figure 4B).

## Discussion

Participants actively explored the environment by using The vOICe (audition) to learn the position of six images (a triangle, a circle, etc.); unlike experiments using vision (e.g., Mou and McNamara, 2002), before the main experiment it was useful familiarize participants with the soundscapes of a triangle, a circle, etc. After the training, in the main experiment participants used The vOICe to explore the scene in an egocentric manner, thus emphasizing spatial relationships among the “observer” and the images. Then a map was provided to investigate whether it could trigger spatial updating as in Pasqualotto et al. (2013b). Even though participants egocentrically learnt the locations of the images, the results of the JRD task suggested that the scene was allocentrically represented (Pasqualotto et al., 2013b). This suggests that the egocentric auditory scene was *updated* into an allocentric representation by somatosensory information (map), like visual scenes were updated by somatosensory information generated by self-motion (Farrell and Thomson, 1998; Burgess et al., 2004). Spatial updating has been extensively studied for single objects (Woods and Newell, 2004; Newell et al., 2005) as well as for multiple objects (Diwadkar and McNamara, 1997; Simons and Wang, 1998; Waller et al., 2002; Mou et al., 2004); visual and somatosensory modalities were also investigated (Pasqualotto et al., 2005; Mou et al., 2006; Zhao et al., 2007). Since both vision and somatosensation are well-suited to convey information about shape and the position of objects (at least within the peripersonal space), they have received substantial attention (Ballesteros et al., 1998; Kappers and Koenderink, 1999). Contrarily, spatial updating in audition is little studied because audition is well-suited for localizing objects that emit sounds (see Ho and Spence, 2005; Yamamoto and Shelton, 2009), but not for silent objects (i.e., the vast majority of the objects). We overcame this limit by employing The vOICe, which uses audition to convey information about the shape and the location of objects.

Our results showed that spatial updating occurs for actively learnt auditory scenes and, combined with previous findings on vision and somatosensation (Ballesteros et al., 1998; Avraamides et al., 2004; Lacey et al., 2007; Giudice et al., 2011), suggest that spatial representation is independent from the sensory modality used to explore the space. In other words, our results suggest that visual, somatosensory and auditory spatial information generates equivalent spatial representations. These findings are corroborated by studies showing how spatial information conveyed by different modalities is processed in the same brain areas; one of them is the posterior parietal cortex (PPC, Sakata and Kusunoki, 1992; Knudsen and Brainard, 1995; Farrell and Robertson, 2000; Makin et al., 2007; Morris et al., 2007). In fact, it has been suggested that visual, auditory, and somatosensory information is initially processed by the respective primary sensory cortices (e.g., visual information is processed by primary visual cortex) before being conveyed to “higher level” cortices via two different pathways specialized for identity and location of objects (Mishkin et al., 1983; Lomber and Malhotra, 2008). For all sensory modalities, the pathways specialized for object localization reach the posterior parietal cortex, which processes spatial information disregarding the modality that generated it (Anderson, 2010; Kandel et al., 2012). Areas processing spatial information arising from different sensory modalities include also the prefrontal cortex and the hippocampus (Ghazanfar and Schroeder, 2006; Avenanti et al., 2012; Hartley et al., 2014), and in recent times it has been found that multisensory processing occurs also in areas believed to be strictly unisensory, such as primary sensory cortices (Pascual-Leone and Hamilton, 2001; Sadato et al., 2007; Beer et al., 2011; Pasqualotto et al., 2015). Although speculative, this neuroscientific evidence can explain the results we obtained in our experiment.

An alternative explanation of our results is that, although vision was not involved, our participants created mental images of the scene. There are numerous empirical findings showing that mental imagery is largely visually based (Arditi et al., 1988; De Volder et al., 2001; Tokumaru et al., 2003; Ganis et al., 2004), therefore it is possible that our participants created visual images of the unseen scene. Recoding non-visual spatial information into visual images might explain the finding of the current study (using audition) and of other studies using non-visual modalities for spatial exploration (Yamamoto and Shelton, 2009; Avraamides and Kelly, 2010; Schifferstein et al., 2010). The role of visual mental imagery could be tested in future experiments where individuals without visual experience, and thus without visual imagery, are tested (i.e., congenitally blind participants; for a review see Pasqualotto and Proulx, 2012). Another idea for future studies is to employ The vOICe for allocentric spatial learning rather than egocentric. In the present study we used egocentric learning (images were learnt one-by-one by following a sequence), but in principle it is possible to use the sensory substitution device to explore the *entire* scene (and not image-by-image), thus emphasizing the spatial relations among objects (i.e., allocentrically). As a matter of fact, in our lab we have already started working on the latter idea; preliminary and possibly “temporary” results are suggesting that participants can learn auditory scenes (through The vOICe) with a level of accuracy comparable to visual scenes.

It is particularly important to note that in a previous study using the same methods, but involving somatosensation (Pasqualotto et al., 2013b), blindfolded sighted participants produced a much poorer performance than in the present study. Although the pattern of the results was equivalent (i.e., allocentric representation displayed by a saw-tooth pattern), the overall performance was more accurate in this study using sensory substitution. This supports the ability of The vOICe to successfully convey spatial information. Our results showed that information acquired though visual-to-auditory sensory substitution (The vOICe) can be updated by when allocentric information is provided; this finding has practical implications, because training regimens with sensory substitution could be improved by providing allocentric spatial information—as we did by using a map. Problems connected to long trainings with SSD, or the absence of training protocols, have been identified as major obstacle for the use of these tools in real-world settings (Loomis, 2010; Maidenbaum et al., 2014).

Our participants were able to update the representation of the scene from egocentric to allocentric (i.e., achieve a more “global” representation). Yet, this finding needs to be confirmed by testing visually impaired individuals. In fact, there is convincing evidence that congenitally blind (individuals with no visual experience) find particular problematic to achieve allocentric spatial representation (Putzar et al., 2007; Pasqualotto and Proulx, 2012; Gori et al., 2014).

In sum, our study offers a *potential* new avenue for reducing the number of training sessions necessary for using The vOICe in real-world settings and it could help a conspicuous portion of visually impaired individuals to improve their mobility and social interactions (Dundon et al., 2015).

## Author Contributions

AP followed every phase of the work; TE contributed to collect data and to discuss/interpret the results.

## Conflict of Interest Statement

The authors declare that the research was conducted in the absence of any commercial or financial relationships that could be construed as a potential conflict of interest.

## Footnotes

^1^Throughout the manuscript, the term “image” will be used for both visual and sonified images and the context will clarify its exact meaning.

^2^Although the terms “viewpoint”, “observer”, etc. are connected to the visual modality, for sake of consistency and clarity we will continue to use them when scenes were explored through non-visual modalities.

^3^Not the area of the floor where images were located.

^4^For the egocentric condition skewness was 0.048 (SE = 0.536) and a kurtosis was −1.736 (SE = 1.038); for the allocentric condition skewness was 0.820 (SE = 0.536) and kurtosis was −0.08 (SE = 1.038; Cramer, 1998).

^5^For the egocentric condition skewness was 1.286 (SE = 0.536) and a kurtosis was 2.035 (SE = 1.038); for the allocentric condition skewness was 1.120 (SE = 0.536) and kurtosis was 1.910 (SE = 1.038).
